# Restriction-deficient mutants and marker-less genomic modification for metabolic engineering of the solvent producer *Clostridium saccharobutylicum*

**DOI:** 10.1186/s13068-018-1260-3

**Published:** 2018-09-27

**Authors:** Ching-Ning Huang, Wolfgang Liebl, Armin Ehrenreich

**Affiliations:** Chair of Microbiology, Technical University of Munich, Freising, 85350 Germany

**Keywords:** 5-Fluorocytosine, *CodB/codA*, Xylulose kinase, Butyrate kinase, Phosphotransbutyrylase conjugation

## Abstract

**Background:**

*Clostridium saccharobutylicum* NCP 262 is a solventogenic bacterium that has been used for the industrial production of acetone, butanol, and ethanol. The lack of a genetic manipulation system for *C. saccharobutylicum* currently limits (i) the use of metabolic pathway engineering to improve the yield, titer, and productivity of *n*-butanol production by this microorganism, and (ii) functional genomics studies to better understand its physiology.

**Results:**

In this study, a marker-less deletion system was developed for *C. saccharobutylicum* using the *codBA* operon genes from *Clostridium ljungdahlii* as a counterselection marker. The *codB* gene encodes a cytosine permease, while *codA* encodes a cytosine deaminase that converts 5-fluorocytosine to 5-fluorouracil, which is toxic to the cell. To introduce a marker-less genomic modification, we constructed a suicide vector containing: the *catP* gene for thiamphenicol resistance; the *codBA* operon genes for counterselection; fused DNA segments both upstream and downstream of the chromosomal deletion target. This vector was introduced into *C. saccharobutylicum* by tri-parental conjugation. Single crossover integrants are selected on plates supplemented with thiamphenicol and colistin, and, subsequently, double-crossover mutants whose targeted chromosomal sequence has been deleted were identified by counterselection on plates containing 5-fluorocytosine. Using this marker-less deletion system, we constructed the restriction-deficient mutant *C. saccharobutylicum ΔhsdR1ΔhsdR2ΔhsdR3*, which we named *C. saccharobutylicum* Ch2. This triple mutant exhibits high transformation efficiency with unmethylated DNA. To demonstrate its applicability to metabolic engineering, the method was first used to delete the *xylB* gene to study its role in xylose and arabinose metabolism. Furthermore, we also deleted the *ptb* and *buk* genes to create a butyrate metabolism-negative mutant of *C. saccharobutylicum* that produces *n*-butanol at high yield.

**Conclusions:**

The plasmid vectors and the method introduced here, together with the restriction-deficient strains described in this work, for the first time, allow for efficient marker-less genomic modification of *C. saccharobutylicum* and, therefore, represent valuable tools for the genetic and metabolic engineering of this industrially important solvent-producing organism.

**Electronic supplementary material:**

The online version of this article (10.1186/s13068-018-1260-3) contains supplementary material, which is available to authorized users.

## Background

*Clostridium saccharobutylicum* NCP 262 is a solventogenic strain that has been used in South Africa for the industrial production of acetone, butanol, and ethanol (ABE) by fermentation [1, 2]. *C. saccharobutylicum* contains the three type I restriction–modification systems (*hsdR1*: CLSA_RS02150, *hsdR2*: CLSA_RS14125, and *hsdR3*: CLSA_RS04425), which might be why it is so difficult to transform. An efficient tri-parental mating system that transfers in vivo methylated DNA [3] by conjugation has, therefore, been developed to prevent DNA restriction and facilitate the genetic engineering of *C. saccharobutylicum* [4]. Type I restriction–modification (RM) systems consist of three genes, *hsdR*, *hsdM,* and *hsdS*, encoding a restriction enzyme, a methyltransferase, and a specificity subunit, respectively [5]. A restriction-less, marker-less mutant of *Clostridium acetobutylicum* [6] was previously constructed that greatly facilitates the development of reverse genetic tools for this organism. This mutant will also be useful for functional genomics studies and the efficient genetic and metabolic engineering of *C. saccharobutylicum.*

To date, most of the knockout mutants of solventogenic clostridia have been constructed by inserting a group II intron [7–9] or an antibiotic resistance cassette into, or in place of, the genes of interest [10–13]. In these cases, persisting DNA sequences such as an intron, an FRT (Flippase Recognition Target), or resistance markers remain in the strain, and are accompanied by polar effects on the expression of downstream genes [14]. Thus, methods that facilitate the generation of marker-less in-frame deletions in solventogenic clostridia are necessary. Moreover, another advantage of such methods is that they can introduce multiple knockouts or insertions, since the number of available resistance markers is not limiting. Typical marker-less deletion systems are two-step methods. First, a non-replicative plasmid containing an antibiotic resistance marker for selecting the allele regions of the target gene is integrated into the bacterial genome by homologous recombination. Then, the vector is excised in a second homologous recombination and selected for using a conditionally lethal counterselection marker present on the plasmid to yield either the wild-type or desired mutant genotype.

Counterselection strategies utilizing the *sacB* system have been used in several Gram-negative bacteria for this purpose, but do not work satisfactorily in most Gram-positive bacteria [13, 15]. Commonly used approaches for counterselection in Gram-positive bacteria exploit either endogenous toxin/antitoxin systems such as *mazE/mazF* [16–18] or gene-encoding enzymes involved in the purine or pyrimidine metabolism. For example, *upp* (phosphoribosyltransferase), *codA* (cytosine deaminase) [19, 20], *pyrE*/*ura5* (orotate phosphoribosyltransferase), and *hpt* (hypoxanthine phosphoribosyltransferase) have all been used [20–26]. All these exemplary systems are based on the same selection principle, i.e., that purine or pyrimidine analogs are converted to toxic compounds and that cells can only survive in the presence of the analog when they lack the gene for the converting enzyme. In a previous study by our group, the *upp* gene was utilized for the counterselection step [27]. The uracil phosphoribosyltransferase encoded by this gene catalyzes the conversion of the pyrimidine analog 5-fluorouracil (5-FU) to 5-fluorouridine-monophosphate [28]. This is then transformed to 5-fluorodesoxyuridine-monophosphate, which elicits a toxic effect by inhibition of thymidylate synthase, thereby blocking DNA repair and replication [29]. Counterselection against this vector was, therefore, performed on media supplemented with 5-FU. In spite of this system’s high efficiency, the requirement for using a Δ*upp* strain limits its application in a variety of solventogenic clostridia used in biotechnology. Cytosine deaminase is an enzyme that participates in pyrimidine salvage metabolism by catalyzing the deamination of cytosine to uracil, but it can also convert the cytosine analog 5-fluorocytosine (5-FC) to 5-FU [30]. A cytosine deaminase system has been used for a negative selection procedure in *Streptomyces* species and *Rhodococcus equi* [31], while 5-FC has been used for negative selection conferred by a heterologously expressed *E. coli codA* gene in mammalian cells and several Gram-positive bacteria [32–35]. Recent approaches also include the use of the CRISPR/Cas9 systems for counterselection, because the induced double strand breaks in the target gene are lethal in prokaryotes [36–38]. In this study, we report the use of the *codBA* operon genes derived from *C. ljungdahlii* as counterselection markers in combination with 5-FC as the counterselective compound for the generation of marker-less chromosomal deletions in the Gram-positive species *C. saccharobutylicum*. This method was used to generate marker-less restriction-deficient mutants of *C. saccharobutylicum*. In addition, the *xylB* gene was deleted to study the role of its encoded carbohydrate kinase in xylose and arabinose metabolism and a butyrate metabolism-negative strain that produces *n*-butanol at high yield was also produced by deletion of the *ptb* and *buk* genes.

## Results

### Generation of the Δ*hsdR1* strain, the first marker-less *C. saccharobutylicum* strain that is transformable without prior in vivo plasmid methylation

**Fig. 1:**
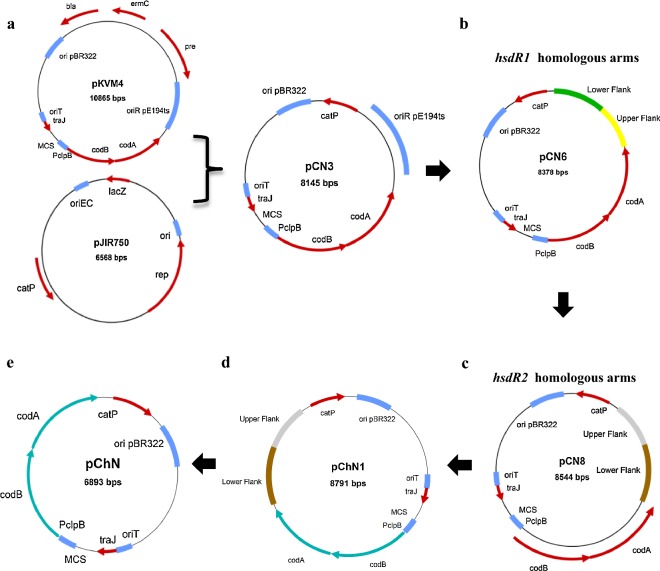
Schematic representation of deletion vector construction. **a** pCN3, a shuttle vector for *C. saccharobutylicum* NCP262 in which the antibiotic cassette of pKVM4 is replaced by the *catP* gene from pJIR750. **b** pCN6, a suicide vector to delete the *hsdR1* gene, where the pE194ts replicon is replaced by *hsdR1* homologous arms. **c** pCN8, where the homologous arms of pCN6 are replaced by those *hsdR2*. **d** pChN1, a deletion vector for the *hsdR2* where the *codBA* operon genes of pCN8 are replaced by those from *C. ljungdahlii*. **e** pChN, a deletion vector cassette produced by removing the *hsdR2* homologous arms from pChN1

**Fig. 2:**
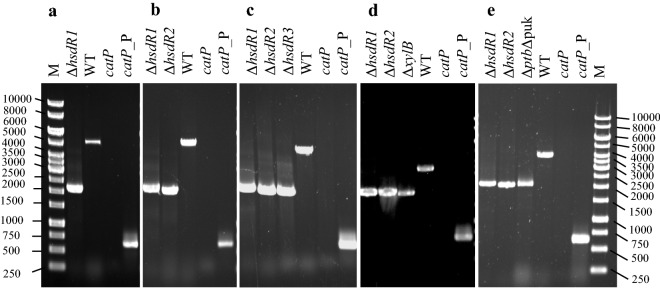
Gene replacement via allelic exchange at the *hsdR1*, *hsdR2*, *hsdR3*, *xylB,* and *ptb*–*buk* loci. PCR confirmation of the different double-crossover deletion mutants using external primers annealing to the chromosome upstream and downstream of each deletion cassette. Strains (**a**) Δ*hsdR1*. **b** Δ*hsdR1* Δ*hsdR2*. **c** Δ*hsdR1* Δ*hsdR2* Δ*hsdR3*. **d** Δ*hsdR1* Δ*hsdR2* Δ*xylB*. **e** Δ*hsdR1* Δh*sdR2* Δ*ptb* Δ*buk*. Δ*hsdR1*: 2141 bp (**a**, **b**, **c**, **d**, **e**), WT of *hsdR1*: 5553 bp (**a**), *catP* gene: 622 bp (**a**, **b**, **c**, **d**, **e**). Δ*hsdR2*: 2064 bp **(b**, **c**, **d**, **e**), WT of *hsdR2*: 5259 bp (**b**) Δ*hsdR3*: 2078 bp (**c**), WT of *hsdR3*: 5010 bp (**c**). Δ*xylB*: 2081 bp (**d**), WT of *xylB*: 3549 bp (**d)**. Δ*ptb* Δ *buk*: 2042 bp (**e**), and WT of *ptb*–*buk*: 4026 bp (**e**)

The genome of the biotechnologically important solventogenic *Clostridium saccharobutylicum* NCP 262 contains three operons coding for genes of presumed type I RM systems belonging to the families A and C. The first RM system (RM1) consists of three genes, *hsdR1, hsdM1,* and *hsdS1,* encoding the restriction, methylation, and specificity subunits, respectively. Similarly, the second (RM2) and third RM (RM3) systems are composed of the *hsdR2, hsdM2,* and *hsdS2* and the *hsdR3, hsdM3,* and *hsdS3* genes, respectively. The previous work in our laboratory aimed at determining the importance of RM1 and RM2 in the restriction of exogenous DNA introduced into *C. saccharobutylicum*, resulted in the generation of the *hsdR1*::int ClosTron mutant. This strain was used to prevent exogenous DNA from degradation by both restriction systems by introducing (by conjugation) recombinant DNA that had been previously methylated in vivo for protection against degradation by RM2 [4]. Furthermore, we constructed a vector suitable for counterselection in *C. saccharobutylicum* using the *codBA* operon genes from *E. coli* K12 that encode a cytosine transporter (*codB*) and a cytosine deaminase (*codA*). These two genes have been successfully used by us as a counterselection marker in combination with 5-FC as the counterselective compound in the Gram-positive bacterium *Bacillus licheniformis* [34]. The *hsdR1*::int gene was deleted using a suicide vector carrying the replacement cassette, which was constructed in two steps. First, the pCN3 vector was produced by replacing the *bla*, *ermC,* and the *pre* genes from pKVM4 by the *catP* gene from pJIR750 (Fig. 1a). Then, an upstream and a downstream flanking region of the target *hsdR1* gene were amplified (each region about 1 kb), fused, and inserted into pCN3 in place of the Gram-positive pE194ts replicon to yield the suicide vector pCN6 (Fig. 1b). After in vivo methylation using *E. coli,* Top 10 containing pJL2, pCN6 was introduced into the *C. saccharobutylicum hsdR1*::int strain by tri-parental conjugation. The transconjugants were plated on 2×YTG plates supplemented with 15 μg/ml thiamphenicol and 10 μg/ml colistin (for selection against *E. coli* cells used in tri-parental mating) and incubated overnight at 37 °C under anaerobic conditions. PCR showed that clones resistant to thiamphenicol were the result of homologous recombination of pCN6 with either the upstream or the downstream region of *hsdR1* on the *C. saccharobutylicum hsdR1*::int strain chromosome. Colonies were streaked on MES-MM plates containing 0.01% yeast extract and 60–600 μg/ml of 5-FC, to select clones that have lost the *codBA* operon genes after a second crossover. However, after overnight incubation at 37 °C, all the colonies obtained were still resistant to thiamphenicol when tested by replica plating. Furthermore, colony PCR analysis showed that the *catP* gene was still present and that the colonies contained a mix of single integrants comprising cells of the *hsdR1*::int strain and Δ*hsdR1* mutants. This suggested that the 5-FC selection did not function optimally, perhaps, because the *codBA* operon was not well expressed. To isolate a Δ*hsdR1* mutant, a colony, giving, after PCR, a high amount of amplification product specific for Δ*hsdR1,* was picked and plated on MES-MM plates containing 0.01% yeast extract, and around 400 colonies were replica plated on the same medium supplemented with 5 μg/ml erythromycin. Among these, two clones were erythromycin-sensitive and, when analyzed by PCR, were shown to be Δ*hsdR1* mutants (Fig. 2a).

### Construction of a *C. saccharobutylicum* Δ*hsdR1*Δ*hsdR2* strain using the *codB*–*codA* genes from *C. ljungdahlii*

Since 5-FC counterselection was suboptimal, we assumed that the *codBA* operon genes from *E. coli* were not sufficiently well expressed in *C. saccharobutylicum,* and consequently, we decided to construct a new suicide vector, pChN1, using the *codBA* operon genes from *Clostridium ljungdahlii* to delete *hsdR2*. First, upstream and downstream flanking regions of the target *hsdR2* gene were amplified (each region about 1 kb), fused, and inserted into pCN6 in place of the *hsdR1* deletion cassette to yield pCN8 (Fig. 1c). Then, the *codBA* operon genes from *E. coli* were replaced by their clostridial orthologs (CLJU_RS09415 and CLJU_RS09420) from *C. ljungdahlii* (Fig. 1d). After in vivo methylation against HsdR2 restriction using pJL2, pChN1 was introduced into the *C. saccharobutylicum* Δ*hsdR1* strain by tri-parental conjugation as described by Lesiak et al. [4]. The transconjugants were then plated on 2×YTG supplemented with 15 μg/ml thiamphenicol and 10 μg/ml colistin (for selection against *E. coli* cells in the conjugation mix) and incubated overnight at 37 °C under anaerobic conditions. PCR showed that the clones resistant to thiamphenicol were the result of homologous recombination of pChN1 with either the upstream or the downstream region of *hsdR2* on the chromosome of the *C. saccharobutylicum* Δ*hsdR1* strain. Colonies were then streaked and grown overnight on MES-MM plates supplemented with 0.001% yeast extract and 500 μg/ml of 5-FC to select for clones that had lost the *codBA* operon genes by a second crossover (Fig. 2b).

The colonies were then replica plated on the same medium and on MES-MM plates containing 0.001% yeast extract and 15 μg/ml of thiamphenicol. Twenty colonies that did not grow on the thiamphenicol plate were analyzed by PCR for *hsdR2* deletion. About half (9 of 20) possessed the desired genotype (i.e., deletion of *hsdR2*), while the remainder were wild type. This demonstrates that the *codBA* operon genes from *C. ljungdahlii* were functionally expressed in *C. saccharobutylicum* and that they can be used in combination with 5-FC for counterselection. The resulting *C. saccharobutylicum* Δ*hsdR1*Δ*hsdR2* strain, which we named *C. saccharobutylicum* Ch1, was further used to construct a restriction-deficient strain by deletion of the *hsdR3* gene.

### Construction of *C. saccharobutylicum* Δ*hsdR1*Δ*hsdR2*Δ*hsdR3,* a restriction-minus strain that can be subjected to iterative genome modification without marker limitations

Based on the success of the *hsdR2* deletion using the pChN1 deletion vector and the *codBA* operon genes from *C. ljungdahlii* for counterselection, we used pChN1 as a backbone to construct a generic deletion vector, pChN, lacking homologous arms (Fig. 1e). About 1 kb of the upstream and downstream flanking regions of the target *hsdR3* gene were amplified, fused, and inserted into pChN to produce the pChN2 plasmid. This plasmid was introduced into the *C. saccharobutylicum* Ch1 strain by tri-parental conjugation [4] without prior in vivo methylation. A clone with a deletion in *hsdR3* was selected, as described above for *hsdR2* (Fig. 2c), to produce the *C. saccharobutylicum* Δ*hsdR1*Δ*hsdR2*Δ*hsdR3* strain, which we named *C. saccharobutylicum* Ch2.

The unmethylated plasmid pMTL84151 was used to evaluate the conjugation efficiency of the *C. saccharobutylicum* wild type, Δ*hsdR1,* Ch1 and Ch2 strains. As reported previously [4], no transconjugants could be observed in the wild-type strain without prior in vivo methylation of the plasmid. In contrast, the conjugation efficiencies of the Ch1 and Ch2 strains using unmethylated pMTL84151 were twofold and tenfold higher, respectively, than the Δ*hsdR1* strain (Table 1).

**Table 1:** Transconjugation efficiencies with unmethylated pMTL84151 donor plasmid

*C. saccharobutylicum* strain	Conjugation efficiency with unmethylated pMTL84151
WT	0
*ΔhsdR1*	3.2 ± 0.7 × 10^−4^
Ch1	6.8 ± 1.1 × 10^−4^
Ch2	3.7 ± 1.5 × 10^−3^

Transconjugation efficiencies were calculated as the ratio of colonies on colistin plates with and without thiamphenicol. Mean values and standard deviations from three independent experiments are given

The fermentation profiles of the different strains were evaluated in batch fermentation performed without pH regulation in MS medium. Solvent and acid formation by *C. saccharobutylicum* Ch1 were similar to the wild-type strain (Table 2), indicating that no physiological modifications were introduced during the construction of the mutants.

**Table 2:** Solvent and acid formation by *C. saccharobutylicum* wild-type and mutant strains in batch culture without pH regulation

	Wild type	Ch1	Ch1 Δ*ptb*Δ*buk*
[Acetone]_final_ (mM)	35 ± 2	29.5 ± 1.5	22 ± 1
[Butanol]_final_ (mM)	87 ± 4	81 ± 3	76.5 ± 1.5
[Ethanol]_final_ (mM)	12.5 ± 1.5	10.5 ± 0.5	17.5 ± 0.5
[Acetate]_final_ (mM)	10.5 ± 0.5	13 ± 1	16 ± 1
[Butyrate]_final_ (mM)	13 ± 1	16 ± 1	4.5 ± 1.5
Butanol yield (g·g^−1^)	0.165 ± 0.005	0.155 ± 0.005	0.215 ± 0.005

Mean values and standard deviations from two independent experiments are given

### Application of 5-FC counterselection using the pChN plasmid in *C. saccharobutylicum* Ch1 to study the role of the *xylB* carbohydrate kinase gene in xylose and arabinose metabolism

**Fig. 3:**
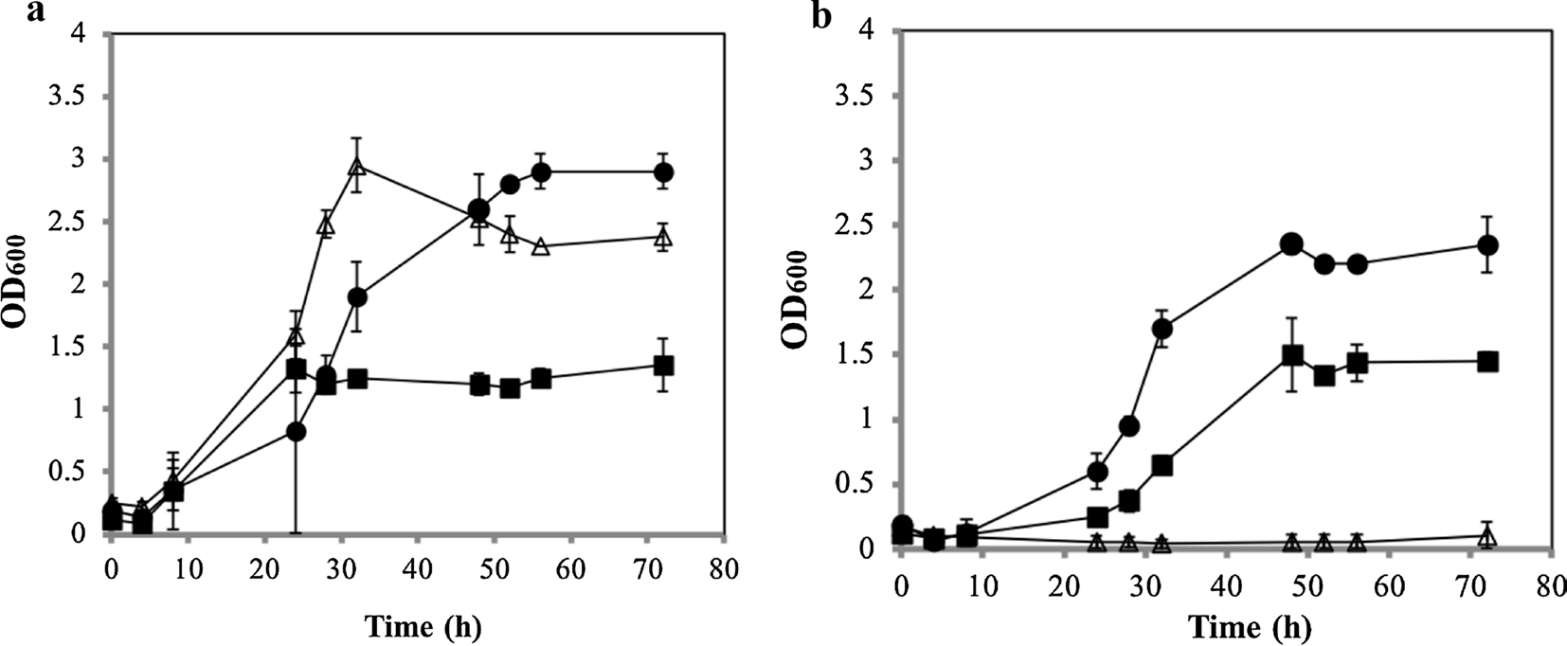
Growth of *C. saccharobutylicum* Ch1 (**a**) and *C. saccharobutylicum* Ch1 Δ*xylB* (**b**) on different carbon sources. Cells were grown in 30 ml of MES-MM supplemented with 0.001% yeast extract and 40 g/l d-glucose (black circle), 40 g/l l-Arabinose (black square) or 40 g/l d-xylose (white up-pointing triangle)

*Clostridium saccharobutylicum* possesses an operon, CLSA_RS15825-CLSA_RS15800, containing six genes potentially involved in xylose metabolism and predicted to code for (1) carbohydrate kinase (*xylB*), (2) ROK family transcriptional regulator, (3) fructose-6-phosphate aldolase, (4) transketolase, (5) DUF4867 family protein, and (6) l-fucose isomerase, with a promoter region-mapped upstream of the CLSA_RS15825 gene. Since the triple-restriction-minus strain was not available at the time of these experiments, the Ch1 double mutant was used as the parental strain. To delete the *xylB* gene from *C. saccharobutylicum* Ch1, pChN3 was constructed from pChN. About 1 kb each of the upstream and downstream flanking regions of the *xylB* gene was amplified, fused, and inserted into pChN to produce the pChN3 plasmid. This plasmid was then introduced into *C. saccharobutylicum* Ch1 by tri-parental conjugation [4] without prior in vivo methylation. Strains with a deletion in the *xylB* gene were selected as described above for *hsdR2* (Fig. 2d). Growth of *C. saccharobutylicum* Ch1 and *C. saccharobutylicum* Ch1 Δ*xylB* on MES-MM liquid cultures supplemented with 0.001% yeast extract or with d-glucose, d-xylose or l-arabinose as sole carbon sources was evaluated. While *C. saccharobutylicum* Ch1 grew on all three carbon sources (Fig. 3a), *C. saccharobutylicum* Ch1 Δ*xylB* only grew on glucose and arabinose but not on xylose (Fig. 3b). This demonstrates that XylB is specifically required for xylose but not for arabinose metabolism.

### Application of 5-FC counterselection using the pChN plasmid for metabolic engineering using the *C. saccharobutylicum* Ch1 strain: deletion of the *ptb*–*buk* operon to create a strain with increased *n*-butanol production

The *ptb* and *buk* genes were targeted for deletion to test the applicability of 5-FC counterselection using the pChN plasmids to the metabolic engineering of *C. saccharobutylicum*. The *ptb* and *buk* genes, which encode a phosphotransbutyrylase and a butyrate kinase, respectively, have been targets for gene inactivation in *C. acetobutylicum,* because the butyrate synthesis pathway competes with the butanol synthesis pathway [39], since the consumption of butyryl-CoA for butyrate formation reduces *n*-butanol yield. The pChN4 vector was, therefore, constructed to delete the *ptb*–*buk* operon from the *C. saccharobutylicum* Ch1 mutant. About 1 kb of sequence upstream and a downstream of the target *ptb*–*buk* operon were amplified, fused, and inserted into pChN to produce the pChN4 plasmid, which was then introduced into the *C. saccharobutylicum* Ch1 strain by tri-parental conjugation [4] without prior in vivo methylation. Clones with a deletion of the *ptb*–*buk* operon were selected as described above for *hsdR2* (Fig. 2e).

The fermentation profile of the *C. saccharobutylicum* Ch1*∆ptb*–*buk* strain was compared to that of the *C. saccharobutylicum* Ch1 control strain in batch fermentation performed without pH regulation in MS medium. The formation of butyrate was highly decreased in the mutant strain and the yield of *n*-butanol on glucose increased from 0.155 to 0.215 g/g (Table 2).

## Discussion

A simple and efficient method to introduce targeted mutations without leaving behind marker remnants in the chromosome was established for *Clostridium saccharobutylicum*.

This method needs: (i) a suitable conjugative suicide shuttle vector; (ii) a deletion cassette containing fused upstream and downstream flanking regions of the target gene; (iii) an efficient counterselection marker, namely the *codBA* operon genes from *Clostridium ljungdahlii*. The *codBA* operon genes encode a cytosine permease and a cytosine deaminase facilitate the conversion 5-FC to 5-FU, which is toxic to the cell. The initial attempts to use the *codBA* operon genes from *E. coli* were unsuccessful, probably because their expression was not codon-optimized for *C. saccharobutylicum* and was, therefore, too low [40]. Other studies have relied on the use of *E. coli codA* alone. However, we have demonstrated before that the additional expression of the gene *codB,* which encodes a cytosine transporter that can presumably transport the cytosine analog 5-FC, enhances the counterselection [34].

The use of suicide plasmids requires high transformation or conjugation efficiencies. This was achieved by employing tri-parental conjugation of *C. saccharobutylicum* with the *E. coli* strains [4] and use of *C. saccharobutylicum* strains with deleted restriction systems. Integration by single crossover was then easily selected for by the thiamphenicol resistance of the clones. The construction of the deletion cassette for the *codBA* operon deletion system for *C. saccharobutylicum* was achieved by fusion PCR based on the SLiCE method. The *codBA* operon genes are located on the pChN plasmid, outside of the deletion cassette. This allows for the positive selection of clones that have lost the plasmid and the integrated deletion cassette via a double recombination event. Once a deletion cassette is integrated into the chromosome, a clean in-frame deletion of the targeted gene can be obtained, thus avoiding polar effects in operon structures. Such strategies were previously applied to construct marker-less gene deletions in *E. coli* [41, 42], *Clostridium difficile* [19], *Bacillus licheniformis* [35], *Gluconobacter oxydans* [34], and many other organisms.

In this study, genes encoding the three type I restriction enzymes of *C. saccharobutylicum*, HsdR1, HsdR2, and HsdR3 (*hsdR1*: CLSA_RS02150, *hsdR2*: CLSA_RS14125, and *hsdR3*: CLSA_RS04425, respectively), were deleted to produce a restriction-deficient strain. The conjugation efficiencies of the *C. saccharobutylicum* Ch1 and *C. saccharobutylicum* Ch2*-*recipient strains using an unmethylated pMTL84151 plasmid, were twofold and tenfold higher than for *C. saccharobutylicum* Δ*hsdR1.* The *C. saccharobutylicum* Ch2 strain should be especially useful for future genetic engineering efforts, e.g., for *mariner* transposon mutagenesis using a suicide vector introduced by conjugation or for the development of a protocol for the transformation of plasmids by electroporation [43]. The *C. saccharobutylicum* Ch1 strain and the *codBA*-based counterselection method described here were successfully used to investigate the role of the putative *xylB* gene in xylose and arabinose metabolism. This work demonstrated that *xylB* encodes a xylulokinase that is essential for the utilization of xylose as a carbon source in *C. saccharobutylicum*.

Furthermore, the described method was successfully used for metabolic engineering by creating a butyrate metabolism-minus strain that produces *n*-butanol at high yield. A similar strain was previously described for *C. acetobutylicum* [39]. A more detailed characterization of the *C. saccharobutylicum* Ch1 Δ*ptb*Δ*buk* growing in both batch and continuous culture is currently in progress in our laboratory.

## Conclusion

The restriction-deficient and marker-less genomic mutants constructed in this study, as well as the associated gene deletion method, will provide, to our scientific community, the simple and convenient tools for the genetic engineering of *C. saccharobutylicum* that can be used for future metabolic engineering of this industrially important strain to enhance the production of chemicals and biofuels.

## Methods

### Bacterial strains, culture and growth conditions, plasmids/oligonucleotides, and tests for 5-FU and 5-FC sensitivity

The bacterial strains and plasmids used in this study are listed in Table 3. Oligonucleotides were obtained from Eurofins MWG GmbH (Ebersberg, Germany) and are listed in Table 4. *C. saccharobutylicum* strains were grown under anaerobic conditions at 37 °C in CGM [44], 2×YTG [4], or MES-MM and MS media with a d-glucose concentration of 50 g/l [45]. Solid media were produced by adding 1.5% agar to the liquid media. Media were supplemented, when required, with the appropriate antibiotic at the following concentrations: erythromycin at 5 μg/ml and thiamphenicol at 15 μg/ml for *C. saccharobutylicum*; kanamycin at 50 μg/ml, chloramphenicol at 25 μg/ml and colistin at 10 μg/ml for *E. coli*. Growth curves in batch cultures were generated in 30 ml modified MES-MM medium supplemented with 0.001% yeast extract and 40 g/l d-glucose (GOPOD Format, K-GLUC, Megazyme, Ireland), or 40 g/l d-xylose (K-XYLOSE, Megazyme, Ireland) or 40 g/l l-arabinose (K-ARGA, Megazyme, Ireland) for 3 days. 5-FU was purchased from Sigma-Aldrich (Steinheim, Germany) and 5-FC from TCI Europe N.V. (Zwijndrecht, Belgium). Both were prepared in water as stock solutions of 10 mg/ml. Minimal inhibitory concentrations of 5-FC and 5-FU were determined in MES-MM [45] supplemented with 1%, 0.1%, 0.01%, or 0.001% yeast extract (see Additional file 1).

**Table 3:** Bacterial strains and plasmids used in this study

Strain or plasmid	Relevant characteristics	References
Bacterial strains		
*E. coli*		
TOP10	*F*- *mcrA Δ(mrr*-*hsdRMS*-*mcrBC) Φ80lacZΔM15 Δ lacX74 recA1 araD139 Δ(araleu)7697 galU galK rpsL (StrR) endA1 nupG*	Invitrogen
DH10B	*F*- *mcrA Δ(mrr*-*hsdRMS*-*mcrBC) Φ80dlacZΔM15 ΔlacX74 endA1 recA1 deoR Δ(ara, leu)7697 araD139 galU galK nupG rpsL λ*-	Invitrogen
CA434	HB101 carrying the IncPb conjugative plasmid, R702, Kan^R^	Purdy et al. [47]
*C. saccharobutylicum*		
NCP262	Wild type	DSMZ^a^
*hsdR1*::int	CLSA_RS02150::intron, *ermB*	Lesiak et al. [4]
Δ*hsdR1*	Δ CLSA_RS02150	This study
Δ*hsdR1*, *hsdR2*::pChN1	Δ CLSA_RS02150, CLSA_RS14125 integration of pChN1	This study
Ch1	Δ CLSA_RS02150 Δ CLSA_RS14125	This study
Ch2	Δ CLSA_RS02150 Δ CLSA_RS14125 Δ CLSA_RS04425	This study
Ch1 Δ*xylB*	Δ CLSA_RS02150 Δ CLSA_RS14125 Δ CLSA_RS15825	This study
Ch1 Δ*ptb*Δ*buk*	Δ CLSA_RS02150 Δ CLSA_RS14125 Δ CLSA_RS01285 Δ CLSA_RS01290	This study
Plasmids		
pJL2	Derived from pACYC184, hsdMSII_T7,_ Tc^R^	Lesiak et al. [4]
pMTL84151	pCD6, Cm^R^	Heap et al. [46]
pKVM4	oripE194ts, oripBR322, *pclpB*, *bla*, *ermC,* oriT, *traJ, codBA* from *E. coli*	Kostner et al. [35]
pJIR750	Cm^R^, lacZ, oripMB1, oripIP404	Bannam and Rood [51]
pCN3	oripE194ts, oripBR322, Cm^R^, oriT, *traJ, codBA* from *E. coli*	This study
pCN6	Δ CLSA_RS02150, oripBR322, Cm^R^, oriT, *traJ, codBA* from *E. coli*	This study
pCN8	Δ CLSA_RS14125, oripBR322, Cm^R^, oriT, *traJ, codBA* from *E. coli*	This study
pChN	oripBR322, Cm^R,^ oriT, *traJ, codBA* gene from *C. ljungdahlii*	This study
pChN1	Δ CLSA_RS14125, Cm^R,^ *codBA* gene from *C. ljungdahlii*	This study
pChN2	Δ CLSA_RS04425, Cm^R,^ *codBA* gene from *C. ljungdahlii*	This study
pChN3	Δ CLSA_RS15825, Cm^R,^ *codBA* gene from *C. ljungdahlii*	This study
pChN4	Δ CLSA_RS01285 Δ CLSA_RS01290, Cm^R,^ *codBA* gene from *C. ljungdahlii*	This study

^a^DSMZ Leibniz Institute DSMZ-German Collection of Microorganisms and Cell Cultures

**Table 4:** Oligonucleotides used for PCR amplification

Primer name	Oligonucleotides sequence	Function
pCN3_V_F	GAAAACTTTTTGCGTGTGACAG	pCN3 backbone
pCN3_V_R	CTGTCAGACCAAGTTTAC	
catp_FpJIR_IV	GTAAACTTGGTCTGACAGACCGTATTTCTACGATGTTT	*catP* gene from pJIR750
catp_RpJIR_IV	CTGTCACACGCAAAAAGTTTTCTTTCGGCAAGTGTTCAAG	
FlankA_F6_IV	GATTACAAACGTTGAAGAAGGAAGGAACTGGTCCAGAAG	*hsdR1* upstream
HsdR1_A_Fu_R	CATTTCTTTAGTTCCCTTCTTAATATTTTCCCCCCTACATTC	
HsdR1_B_Fu_F	GAATGTAGGGGGGAAAATATTAAGAAGGGAACTAAAGAAATG	*hsdR1* downstream
FlankB_R6_IV	CTTGAACACTTGCCGAAAAATGGAGGATTTGCCAATA	
pCN6_V_F	CTTCTTCAACGTTTGTAATC	pCN6/pCN8 backbone
pCN6_V_R	TTTCGGCAAGTGTTCAAG	
HsdR1_check_F	GCAGGAGAAAGGATATGG	*hsdR1* wild type or mutant
HsdR1_check_R	CGATACTCCTGCATATGG	
check_preR	ACACAACCGGCACAAACC	check integration
Check_catp_F	AACTATTTATCAATTCCTGCAATTCGTTTAC	*catP* gene on the deletion vector
Check_catp_R	ATGGTATTTGAAAAAATTGATAAAAATAGTTG	
HsdR2_A_F_IV	CTTGAACACTTGCCGAAAGTGTTAGGTTTAAAGAATAC	*hsdR2* upstream
HsdR2_A_Fu_R	GAATAATTAGGAGGGGATTTGATAATAGTTTAATGGCTATTG	
HsdR2_B_Fu_F	CAATAGCCATTAAACTATTATCAAATCCCCTCCTAATTATTC	*hsdR2* downstream
HsdR2_B_R_IV	GATTACAAACGTTGAAGAAGAAGACTGGGATCGATAGC	
pCLcodBA_F_IV	CTACTTAATTGTGTGTAAGATAAAGAAGAAGACTGGGATCGAT	pChN1 backbone
pCLcodBA_R_IV	CATCAATTACCTCCTAAATTAATAATTAGCTAATTTTCGTTTAATTAT	
CLcodBA_F2	AATTATTAATTTAGGAGGTAATTGATG	*codBA* gene from *C. ljungdahlii*
CLcodBA_R2	TTATCTTACACACAATTAAGTAG	
HsdR2_check_F	GGTGGTTCTACAGCAATCTC	*hsdR2* wild type or mutant
HsdR2_check_R	GCTAAGGACGTTGGATTAGC	
pChN_backbone_F	TACTTAATTGTGTGTAAGATAAGTTTCGGCAAGTGTTCAAGAAG	pChN backbone
pChN_backbone_R	CTTATCTTACACACAATTAAGTAGAAGAAC	
HsdR3_A_F_IV	GTTCTTCTACTTAATTGTGTGTAAGATAAGTGTCTATTCAAGTGCTGTGG	*hsdR3* upstream
HsdR3_A_R_IV	GAAATACAGGGGGTGTTAAC GCTTACAAGACCACAACTAG	
HsdR3_B_F_IV	CTAGTTGTGGTCTTGTAAGC GTTAACACCCCCTGTATTTC	*hsdR3* downstream
HsdR3_B_R_IV	CTTCTTGAACACTTGCCGAAA GCTGCAATAGCAAAATATCG	
pChN_V_F	TTTCGGCAAGTGTTCAAGAAG	pChN2/pChN3/pChN4 backbone
pChN_V_R	CTTATCTTACACACAATTAAGTAGAAGAAC	
HsdR3_check_F	TGCTAAAGTATCGCGGTTGTC	*hsdR3* wild type or mutant
HsdR3_check_R	AGCCGTTCTGAAATTGAACTG	
codBA_CL_R	TATGTGGATGGGGAAGAG	Check integration
xylB_A_F_IV	ACTTAATTGTGTGTAAGATAAG CTAATCCATCCGTTATTG	*xylB* upstream
xylB_A_fu_R2_IV	GTTTATTGATGAGGTATT CTTATCCTAGAATTAAAG	
xylB_B_fu_F2_IV	CTTTAATTCTAGGATAAG AATACCTCATCAATAAAC	*xylB* downstream
xylB_B_R_IV	CTTGAACACTTGCCGAAA TTATTAGATGCTTCTTAG	
xylB_check_F	ATTCTCCCGATGAATTATTG	*xylB* wild type or mutant
xylB_check_R	TCCTTCGTTCAATTAAATC	
PTB_F_IV	TTAATTGTGTGTAAGATAAG ATAAAGCGCCAGTACAGC	*ptb* upstream
PTB_R2_fu_IV	CTTTAGCTTCTTCTTCTCCA TCCTTTAATCTTGATAG	
BUK_R_IV	CTTGAACACTTGCCGAAA ACCTAGTACTCCCTGTTC	*buk* downstream
BUK_F2_fu_IV	CTATCAAGATTAAAGGA TGGAGAAGAAGAAGCTAAAG	
PTB_check_F3	CGGCATTAGTTGTAACTG	*Ptb*–*buk* wild type or mutant
BUK_check_R2	GCTCCACTTGCATTCATC	

### DNA manipulation techniques

Routine molecular biological procedures were performed using the standard protocols [48]. NucleoSpin^®^ Plasmid EasyPure kit (Macherey–Nagel, Germany) was used for plasmid preparation. Genomic DNA from *C. saccharobutylicum* was extracted with an Epicenter MasterPure DNA purification kit (Madison, USA) and DNA purification was performed with a NucleoSpin^®^ PCR clean-UP Gel extraction kit (Macherey–Nagel, Düren, Germany). Cloning was via the SLiCE method, which utilizes easily obtained bacterial cell extracts to assemble multiple DNA fragments into recombinant DNA molecules in a single in vitro recombination reaction [49]. PCR was performed according to the manuals provided for enzymes from Thermo Scientific (Schwerte, Germany). Phire Green Hot Start II DNA polymerase was used for analytical reactions and Phusion High-Fidelity DNA polymerase for amplifications requiring proofreading. TakaRa Bio (Otsu, Shiga, Japan) PrimeSTAR^®^ GXL DNA polymerase was used for the amplification of products ≥ 30 kb in length. Colony PCR [50] was used to screen for mutants or to confirm the integration of a deletion vector into the genome.

### Construction of deletion vectors

PCR primers used in the production of all constructs are listed in Table 4. The pCN3 shuttle vector for *C. saccharobutylicum* and *E. coli* was constructed by replacing the *bla* and *ermC* resistance cassettes of pKVM4 [35] with the *catP* gene from pJIR750 [51] (Fig. 1a). The backbone was amplified using pKVM4 as a template. The *catP* gene fragment was amplified using catp_FpJIR_IV and catp_RpJIR_IV primers and pJIR750 as a template. Cloning was performed using the SLiCE method.

To construct the pCN6 suicide vector for the deletion of the *hsdR1* gene of *C. saccharobutylicum,* the pE194ts Gram-positive origin of replication in pCN3 was replaced by a fragment consisting of fused upstream and downstream flanking regions of the *hsdR1* gene (Fig. 1b). The upstream and downstream flanking regions were amplified using chromosomal DNA from *C. saccharobutylicum* wild type as a template, while the backbone was amplified using pCN3 as a template. Cloning was performed using the SLiCE method. Plasmid integration by single crossover was detected using HsdR1_check_F and Catp_FpJIR_IV primers for 5′ integration and HsdR1_check_R and check_pre_R primers for 3′ integration. After selecting clones that had lost the integrated plasmid containing the *codBA* operon genes via a second crossover event, loss was confirmed using colony PCR. The presence or absence of *catP* was confirmed by PCR.

For construction of the pChN1 suicide vector for deletion of the *hsdR2* gene of *C. saccharobutylicum,* approximately 1 kb of the flanking regions upstream and downstream of the *hsdR2* gene were amplified using chromosomal DNA of *C. saccharobutylicum* wild type as a template, fused, and then inserted into pCN6 in place of the *hsdR1* deletion cassette to produce pCN8 (Fig. 1c). The backbone used was the same as for pCN6. The *codBA* operon genes from *E. coli* were then replaced by the clostridial orthologs (CLJU_RS09415 and CLJU_RS09420) from *Clostridium ljungdahlii*, which were amplified with CLcodBA_F2 and R2 primers and using the chromosomal DNA of wild-type *C. ljungdahlii* as a template. The backbone was amplified using pCN8 as a template and cloning was performed using the SLiCE method (Fig. 1d). Plasmid integration by single crossover was detected using HsdR2_check_F and pCLcodBA_F_IV primers for 5′ integration and HsdR2_check_R and Check_catp_F primers for 3′ integration. After selecting clones that had lost the *codBA* operon genes via a second crossover event, loss was confirmed using colony PCR. The presence or absence of *catP* was confirmed by PCR.

pChN is a generic vector containing the *codBA* operon genes from *C. ljungdahlii* but lacking any homologous arms for a target gene (Fig. 1e). Since pChN1 was successfully used to delete *hsdR2*, we used pChN1 as a template to PCR-amplify the pChN fragment using the pChN_backbone_F and pChN_backbone_R primers. Ligation was performed using the SLiCE method.

For construction of the pChN2 suicide vector for deletion of the *hsdR3* gene, approximately 1 kb flanking regions upstream and downstream of *hsdR3* were amplified using chromosomal DNA of wild-type *C. saccharobutylicum* as a template, fused, and inserted into pChN (Fig. 1e) to produce pChN2. The backbone was amplified using pChN as a template and cloning was performed using the SLiCE method. Plasmid integration via single crossover was detected by PCR using HsdR3_check_F and catp_FpJIR_IV primers for 5′ integration and HsdR3_check_R and codBA_CL_R primers for 3′ integration. After selecting clones that had lost the *codBA* operon genes via a second crossover event, loss was confirmed by colony PCR. The presence or absence of *catP* was confirmed by PCR.

For construction of the pChN3 suicide vector for deletion of the *xylB* gene, approximately 1 kb flanking regions up- and downstream of *xylB* were amplified using chromosomal DNA of wild-type *C. saccharobutylicum* as template, fused, and inserted into pChN (Fig. 1e). The backbone was amplified with pChN as a template and cloning was performed using the SLiCE method. Plasmid chromosomal integration via single crossover was detected by PCR using xylB_check_F and catp_FpJIR_IV for 5′ integration and xylB_check_R and codBA_CL_R primers for 3′ integration. After selecting clones that had lost the *codBA* operon genes via a second crossover, loss was confirmed by colony PCR. The presence or absence of *catP* was confirmed by PCR.

For construction of the pChN4 suicide vector for the deletion of the *buk* and *ptb* genes, an approximately 1 kb region upstream of *ptb* and a second approximately 1 kb region downstream of *buk* were amplified using chromosomal DNA of wild-type *C. saccharobutylicum* as a template, fused, and then inserted into pChN (Fig. 1e) The backbone was amplified using pChN as a template. Cloning was performed using the SLiCE method. Plasmid integration by single crossover was detected by PCR using PTB_check_F3 and catp_FpJIR_IV primer for 5′ integration and BUK_check_R2 and PTB_F_IV primers for 3′ integration. After selecting clones that had lost the *codBA* operon genes via a second crossover event, loss was confirmed by colony PCR. The presence or absence of *catP* was confirmed by PCR.

### Tri-parental conjugation

To conjugate pChN plasmids into *C. saccharobutylicum,* we modified the tri-parental conjugation protocol [4] as follows. *C. saccharobutylicum*-recipient cells in Hungate tube-containing anaerobic 2×YTG medium were heat-shocked at 70 °C for 5 min and then incubated at 37 °C, overnight. Donor cells containing the deletion vector in LB medium-containing chloramphenicol at 25 μg/ml and helper *E. coli* CA434 cells in LB medium-containing 50 μg/ml kanamycin were grown aerobically at 37 °C overnight. Cultures of recipient, donor, and helper cells were then inoculated to an OD600 of 0.1–0.2 and grown to an OD600 of 1 in the respective media described above. One ml each of the donor cells and helper cells were then mixed in the same Eppendorf tube and centrifuged at 6000 rpm at room temperature for 5 min. After washing the cells with 1 ml of phosphate-buffered saline (PBS), the pellet was transferred to an anaerobic chamber. Pellets were resuspended in 200 μl of recipient culture and six drops (about 25 μl per drop) were transferred to 2×YTG plates lacking any antibiotics and incubated overnight at 37 °C. Under anaerobic chamber, the cell mixture was collected from the surface of the agar plate, resuspended in 400 μl of PBS, and plated on 2×YTG plates supplemented with 15 μg/ml thiamphenicol and 10 μg/ml colistin and incubated at 37 °C.

### General procedure for the construction of chromosomal deletion strains of *Clostridium saccharobutylicum* using *codBA* operon-based counterselection

**Fig. 4:**
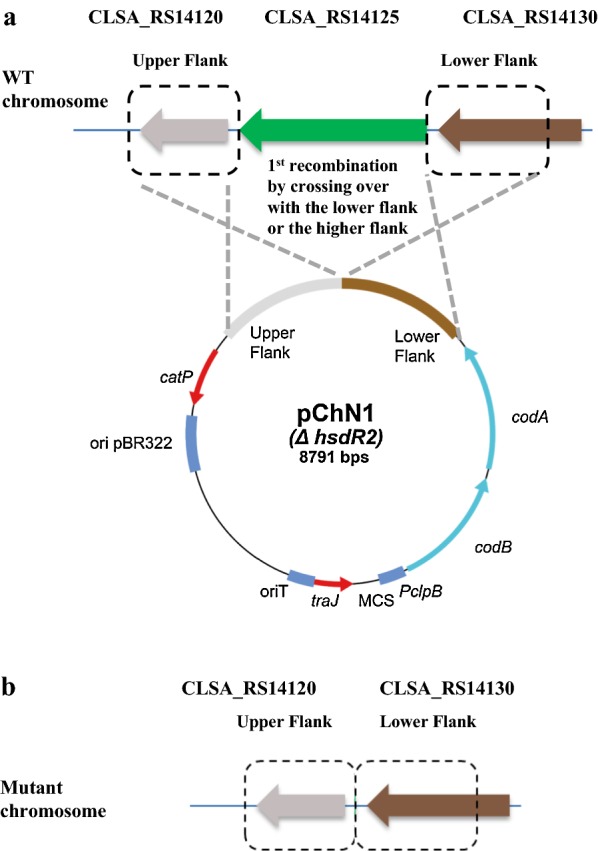
General diagram representing gene replacement via allelic exchange at the target gene. **a** *C. saccharobutylicum* NCP262 genomic regions surrounding CLSA_RS14125 (*hsdR2*). The deletion vector pChN1, containing approximately 1 kbp of upstream and downstream sequences of *hsdR2* and the *codBA* operon from *C. ljungdahlii*. **b** Counterselection strategy with the 5-FC/*codBA* system resulting in a marker-less deletion mutant lacking CLSA_RS14125 (*hsdR2*) between the two flanking regions

The general outline for the deletion method is given below, using the deletion of the *hsdR2* gene from *C. saccharobutylicum* (Fig. 4) as an example. First, a deletion vector containing about 1 kb fused flanking regions from the genomic locus targeted for deletion was constructed. The suicide deletion vector (pChN1 for deletion of *hsdR2*) was methylated by propagation in *E. coli* Top10-containing pJL2 and then introduced into the recipient *C. saccharobutylicum* Δ*hsdR1* by tri-parental conjugation, and with *E. coli* CA434 as a helper strain. Transconjugants are transferred to 2×YTG plates containing 15 μg/ml thiamphenicol for pChN1 selection and 10 μg/ml colistin for elimination of *E. coli*. Since the suicide vector has no functional Gram-positive origin of replication, overnight growth at 37 °C yielded clones with the deletion plasmid integrated into the chromosomal target locus via homologous recombination. Colonies were then picked and streaked on the same medium. The presence of the *catP* gene and integration was confirmed by colony PCR. For counterselection, colonies were streaked on MES-MM supplemented with 0.001% yeast extract containing 500 µg/ml 5-FC, which selected against the vector-encoded *codBA* operon genes. After incubation at 37 °C overnight, only cells that had lost the integrated vector via a second homologous recombination formed colonies. The presence of the expected mutation in the resulting colonies was finally tested by PCR and confirmed by sequencing.

### Analytical methods

Cell growth was monitored by measuring optical density at 600 nm (OD600). Solvent and acid production as well as glucose consumption in cell-free supernatant samples were determined based on high-performance liquid chromatography (HPLC) [52] equipped with refractive index and UV detectors. The separation was obtained with an Aminex HPX-87H (Bio-Rad, Chemical Division, Richmond, USA) column (300 by 7.8 mm). The operating conditions were as follows: temperature, 17 °C; mobile phase, H_2_SO_4_ (0.25 mM); flow rate, 0.5 ml/min [52].

## Additional file

**Additional file 1.** Minimal inhibitory concentration.

## Abbreviations

5-FU: 5-fluorouracil
5-FC: 5-fluorocytosine
CGM: Clostridium growth medium
2×YTG: 2× yeast tryptone glucose medium
PCR: polymerase chain reaction
MES-MM: MES-based mineral medium
RM system: restriction modification system
*catP*: thiamphenicol resistance gene
MS: synthetic mineral medium
SLiCE: seamless ligation cloning extract
