# Supplementary material for: Restriction-deficient mutants and marker-less genomic modification for metabolic engineering of the solvent producer Clostridium saccharobutylicum
Source: Biotechnol Biofuels. 2018 Sep 27;11:264. doi: 10.1186/s13068-018-1260-3 (PMC6158908; doi:10.1186/s13068-018-1260-3)
Supplement: Supplementary file 1 — Additional file 1. Minimal inhibitory concentration. [file 13068_2018_1260_MOESM1_ESM.pdf]

Additional File 1. Minimal inhibitory concentration

Growth of *C. saccharobutylicum* NCP262 on different agar plates; minimal inhibitory concentration of 5-fluorocytosine and 5-fluorouracil.

a

***C. saccharobutylicum* wild type strain**

5-fluorocytosine (µg/ml)	0	30	60	120	240	500
MES-MM	-	-	-	-	-	-
MES-MM + 1%Yeast extract	+	+	+	+	+	+
MES-MM + 0.1%Yeast extract	+	+	+	+	+	+
MES-MM + 0.01%Yeast extract	+	+	+	+	+	+
MES-MM + 0.001%Yeast extract	+	+	+	+	+	+

MES-MM: Mineral Medium MES-based

5-fluorouracil (µg/ml)	0	30	60	120	240	500
MES-MM	-	-	-	-	-	-
MES-MM + 1%Yeast extract	+	-	-	-	-	-
MES-MM + 0.1%Yeast extract	+	-	-	-	-	-
MES-MM + 0.01%Yeast extract	+	-	-	-	-	-
MES-MM + 0.001%Yeast extract	+	-	-	-	-	-

MES-MM: Mineral Medium MES-based

b

***C. saccharobutylicum* (Δ*hsdR1*, *hsdR2*::pChN1) integration strain**

5-fluorouracil (µg/ml)	0	30	60	120	240	500
MES-MM	-	-	-	-	-	-
MES-MM + 1%Yeast extract	+	+	+	+	+	+
MES-MM + 0.1%Yeast extract	+	+	+	+	+	+
MES-MM + 0.01%Yeast extract	+	+	+	+	+	+
MES-MM + 0.001%Yeast extract	+	+	+	+	+	-

MES-MM: Mineral Medium MES-based
